# Listeriolysin O Causes ENaC Dysfunction in Human Airway Epithelial Cells

**DOI:** 10.3390/toxins10020079

**Published:** 2018-02-11

**Authors:** Guang Yang, Helena Pillich, Richard White, Istvan Czikora, Isabelle Pochic, Qiang Yue, Martina Hudel, Boris Gorshkov, Alexander Verin, Supriya Sridhar, Carlos M. Isales, Douglas C. Eaton, Jürg Hamacher, Trinad Chakraborty, Rudolf Lucas

**Affiliations:** 1 Vascular Biology Center, Medical College of Georgia at Augusta University, Room CB-3213B, Augusta, GA 30912-2500, USA; guyang@llu.edu (G.Y.); ICZIKORA@augusta.edu (I.C.); BGORSHKOV@augusta.edu (B.G.); AVERIN@augusta.edu (A.V.); SUSRIDHAR@augusta.edu (S.S.); 2 Institute of Medical Microbiology, Justus-Liebig University Giessen, 35392 Gießen, Germany; helena.pillich@mikrobio.med.uni-giessen.de (H.P.); martina.hudel@mikrobio.med.uni-giessen.de (M.H.); 3 Department of Pharmacology and Toxicology, Medical College of Georgia at Augusta University, Room CB-3213B, Augusta, GA 30912-2500, USA; richardwhit@pcom.edu; 4 Department of Biomedical Sciences, Georgia Campus-Philadelphia College of Osteopathic Medicine, Atlanta, GA 30224, USA; 5 Biochemical Pharmacology, University of Konstanz, 78464 Konstanz, Germany; isabelle.pochic@gmx.de (I.P.); jhamache@hin.ch (J.H.); 6 Sandoz Inc., 83607 Holzkirchen, Germany; 7 Department of Physiology, Emory School of Medicine, Atlanta, GA 30307, USA; qiang.yue@emory.edu (Q.Y.); deaton@emory.edu (D.C.E.); 8 Department of Medicine, Medical College of Georgia, Augusta, GA 30901, USA; CISALES@augusta.edu; 9 Department of Pneumology, Lindenhofspital, 3001 Bern, Switzerland; 10 Internal, Pulmonary and Critical Care Medicine, Saarland University, 66424 Homburg/Saar, Germany; 11 Lungen-und Atmungsstifung, 3001 Bern, Switzerland

**Keywords:** listeriolysin O, TNF, pulmonary permeability edema, epithelial sodium channel, protein kinase C-α

## Abstract

Pulmonary permeability edema is characterized by reduced alveolar Na^+^ uptake capacity and capillary barrier dysfunction and is a potentially lethal complication of listeriosis. Apical Na^+^ uptake is mainly mediated by the epithelial sodium channel (ENaC) and initiates alveolar liquid clearance. Here we examine how listeriolysin O (LLO), the pore-forming toxin of *Listeria monocytogenes*, impairs the expression and activity of ENaC. To that purpose, we studied how sub-lytic concentrations of LLO affect negative and positive regulators of ENaC expression in the H441 airway epithelial cell line. LLO reduced expression of the crucial ENaC-α subunit in H441 cells within 2 h and this was preceded by activation of PKC-α, a negative regulator of the channel’s expression. At later time points, LLO caused a significant reduction in the phosphorylation of Sgk-1 at residue T256 and of Akt-1 at residue S473, both of which are required for full activation of ENaC. The TNF-derived TIP peptide prevented LLO-mediated PKC-α activation and restored phospho-Sgk-1-T256. The TIP peptide also counteracted the observed LLO-induced decrease in amiloride-sensitive Na^+^ current and ENaC-α expression in H441 cells. Intratracheally instilled LLO caused profound pulmonary edema formation in mice, an effect that was prevented by the TIP peptide; thus indicating the therapeutic potential of the peptide for the treatment of pore-forming toxin-associated permeability edema.

## 1. Introduction

The Gram-positive facultative intracellular bacterium *Listeria monocytogenes* causes listeriosis, a severe foodborne disease, characterized by meningitis and meningo-encephalitis. In recent years, an increasing rate of listeriosis has been reported in Europe and the US [1]. High numbers of *Listeria* can be detected in the lung of the newborn in early-onset neonatal listeriosis, most likely due to the uptake of bacteria from contaminated amniotic fluid. This can result in severe pulmonary disease, accompanied by permeability edema, which requires harsh therapeutic measures and often has a fatal outcome [2,3]. Similarly, pleural fluid from severely immune-suppressed patients or from pregnant women can contain significant numbers of *L. monocytogenes* [3]. Thus, although observed rarely in healthy adults, the lung represents a potential port of entry for *L. monocytogenes* in neonates and immune-suppressed individuals. This bacterium evades microbicidal defenses by means of secreting the cholesterol-dependent pore-forming cytolysin and virulence factor listeriolysin O (LLO), which induces a rapid Ca^2+^ influx into mammalian cells [4,5].

Listeriosis-associated permeability edema is characterized by an impaired alveolar-capillary barrier function, in combination with a dysfunctional alveolar liquid clearance (ALC) capacity. ALC is mediated by vectorial sodium transport in type II alveolar and small airway epithelial cells. Na^+^ uptake is mainly regulated by the apically expressed epithelial sodium channel (ENaC), consisting in its main form of α, β, and γ subunits [6,7,8]. A fourth δ subunit was recently demonstrated and can form functional channels with β and γ subunits [9]. The favorable electrochemical driving force for Na^+^ influx is maintained by the basolaterally expressed ouabain-sensitive Na^+^/K^+^-ATPase, which transports Na^+^ into the interstitial space [10]. The surface expression of ENaC is regulated mainly via the neural precursor cell-expressed developmentally downregulated (gene 4) protein (Nedd4-2), which leads to ubiquitination and subsequent degradation of the sodium channel [11]. Specific kinases, such as the cell volume stress-activated serum and glucocorticoid-dependent kinase (Sgk-1) and protein kinase B (Akt-1), control the surface expression of ENaC by means of phosphorylating Nedd4-2 and subsequently reducing its binding to ENaC [12,13,14,15,16]. Alternatively, Sgk-1 has been shown to phosphorylate iNOS in type II alveolar epithelial cells, thereby reducing NO production, an inhibitor of Na^+^ transport [16]. An important negative regulator of ENaC expression is protein kinase C [17,18], which has been shown to be activated by LPS as well as during influenza infection [19,20,21,22].

Spatially distinct from its receptor binding sites, the pro-inflammatory cytokine TNF carries a lectin-like domain, which recognizes specific oligosaccharides, including *N*,*N*′-diacetylchitobiose and branched trimannoses [23]. The lectin-like domain of TNF can be mimicked by a circularized 17 amino acid peptide, the TIP peptide (a.k.a. AP301 and Solnatide) [24]. The lectin-like domain of TNF exerts direct lytic activities toward certain stages of the bloodstream forms of African trypanosomes and *Candida albicans* [24,25,26,27]. In mammalian cells, the TIP peptide increases Na^+^ uptake in A549 cells in a catecholamine-independent manner [28]. The TIP peptide, upon intra-tracheal instillation, was shown to activate ALC in an isolated flooded rat lung model ex vivo, as well as in a flooded rat lung model in situ and in vivo [29,30]. Moreover, the peptide was shown to increase transepithelial current in rat type II alveolar epithelial cells when applied from the apical but not from the basolateral side, indicating its first target of activation is ENaC rather than Na^+^, K^+^-ATPase [31]. Nevertheless, the Na^+^, K^+^-ATPase can also be indirectly activated by the peptide [32]. Recently, the TIP peptide was shown to directly bind to the α subunit of ENaC [33,34], and as such increasing the open probability time *Po* of the channel.

The main aim of this study was to investigate the effect of the main virulence factor of *L. monocytogenes*, LLO, on both positive and negative regulators of ENaC expression, as well as on ENaC expression and function in H441 cell, a human bronchiolar epithelial cell line. Moreover, the effect of LLO on the lung wet-to-dry ratio was assessed in mice. As a second aim, we investigated whether the TIP peptide, mimicking the lectin-like domain of TNF, can interfere with the activities of LLO on ENaC function.

## 2. Results

### 2.1. LLO Decreases ENaC-α Subunit Expression Levels in H441 Cells in a Time-Dependent Manner

**Figure 1:**
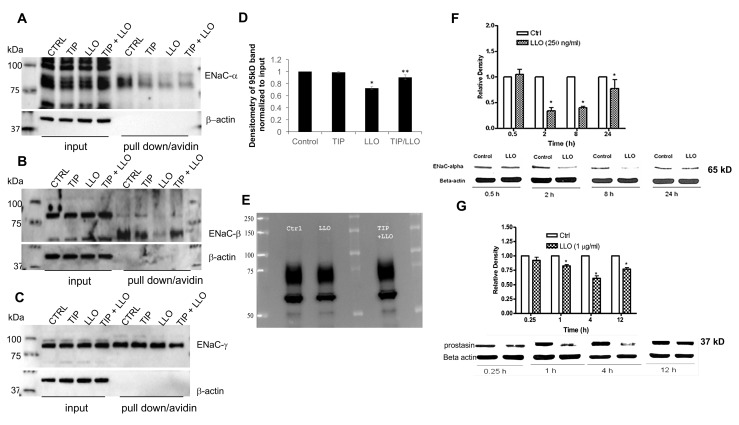
(**A**–**E**) Surface biotinylation experiments at 8 h post listeriolysin O (LLO) treatment (4.3 nM) of (**A**) uncleaved 95 kD epithelial sodium channel (ENaC)-α expression (polyclonal Ab from Novus Biologicals), of (**B**) uncleaved and cleaved ENaC-β (pAb from Protein Tech) and of (**C**) uncleaved ENaC-γ (polyclonal Ab from Abcam) in H441 cells and (**D**) densitometry (from three independent experiments, using the Novus pAb) of uncleaved 95 kD ENaC-α expression in H441 cells treated as above. * *p* < 0.04 vs. ctrl, ** *p* < 0.05 vs. LLO. (**E**) Western blotting of both uncleaved and cleaved ENaC-α expression using a home-made polyclonal Ab [35]. (**F**) Western blotting of mature (65 kD) ENaC-α expression at 0.5, 2, 8, and 24 h post LLO (4.3 nM) treatment (total protein). (**G**) Western blotting of prostasin expression (band around 37 kD) after 0.25, 1, 4, and 12 h of LLO treatment (17.2 nM) in H441 cells. Mean ± SEM, *n* = 3, * *p* < 0.05 vs. ctrl.

Since ENaC subunits, especially ENaC-α, are crucial for ENaC function, we investigated whether LLO can alter the surface expression level of ENaC-α, -β, and -γ in H441 cells, using a surface biotinylation approach. As shown in Figure 1A–C, LLO (4.3 nM) reduced the surface expression of uncleaved ENaC-α, as well as of cleaved and uncleaved ENaC-β in H441 cells, whereas we could not detect a significant reduction in ENaC-γ expression. The reduction in ENaC-α expression was significant, as quantified in Figure 1D. Since some commercial antibodies do not recognize both cleaved and uncleaved forms of the subunits, we also assessed ENaC-α surface expression using a home-made polyclonal antibody [35] that recognizes both the uncleaved and mature forms of the subunit. We detected that LLO treatment reduced the expression of both uncleaved and cleaved ENaC-α in H441 cells within 8 h. The TNF-derived TIP peptide (22 μM), which was previously shown to protect from pneumolysin-induced downregulation of ENaC-α [33], when given to the cells 30 min before LLO, prevented LLO-induced downregulation of ENaC-α (Figure 1A,D,E) and -β expression (Figure 1B). As shown in Figure 1F, LLO-induced reduction in ENaC-α expression was already detectable at 2 h of LLO treatment and was only restored after 24 h (total protein). These results, therefore, indicate that LLO suppresses the surface expression of both mature and uncleaved ENaC-α expression in H441 cells in a time-dependent manner. We, therefore, also evaluated the effects of LLO on the expression levels of the trypsinogen prostasin, a member of the trypsin family of serine proteases, which is involved in the cleavage and maturation of ENaC subunits [36]. As shown in Figure 1G, LLO (17.2 nM) significantly decreased the expression of prostasin (band at around 37 kD) from 1 h of incubation onward with a maximal reduction detected at 4 h.

### 2.2. LLO-Induces PKC-α Activation in H441 Cells

Several studies have shown that LLO can form Ca^2+^-permeable pores, which causes intracellular Ca^2+^ oscillations [4,5]. Increased intracellular Ca^2+^ levels, together with increased levels of diacylglycerol, can induce the activation of conventional PKC isoforms [37]. Conventional PKC isoforms were shown in previous studies to decrease Na^+^ reabsorption across the alveolar epithelium by affecting the function of amiloride-sensitive ENaC [17,38]. In addition, inhibition of PKC activity has been shown to increase sodium channel activity [20,39].

**Figure 2:**
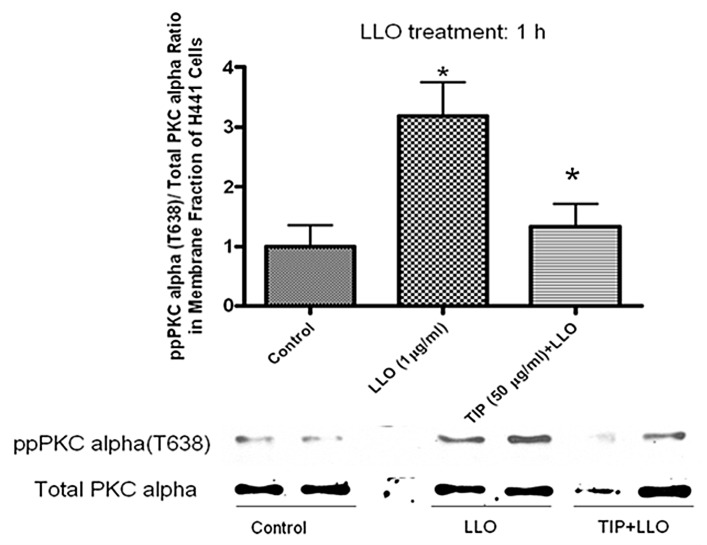
LLO (17.2 nM) significantly induced PKC-α phosphorylation at T638 within 1 h in H441 cells, which causes activation of the enzyme. A 30 min pretreatment with the TIP peptide (50 μg/mL, 22 μM) attenuated LLO-induced phosphorylation of pT638-PKC-α. Mean ± SEM, *n* = 3, * *p* < 0.05 vs. ctrl; ** *p* < 0.05 vs. LLO.

As shown in Figure 2, LLO at a concentration of 17.2 nM leads to a significant activation of PKC-α within 1 h, as measured by the ratio of pT638-PKC-α over total PKC-α in the membrane fraction of the H441 cells. Pretreatment of the cells with the TNF-derived TIP peptide (22 μM)—which was previously shown to activate Na^+^ uptake in alveolar epithelial cells [31]—30 min prior to applying LLO can effectively attenuate LLO-induced phosphorylation of PKC-α at T638, one of its carboxy-terminal sites.

### 2.3. The Protein Kinase C-α Inhibitor Ro-32-0432 Significantly Prevents LLO-Mediated Reduction of ENaC-α Expression in H441 Cells

**Figure 3:**
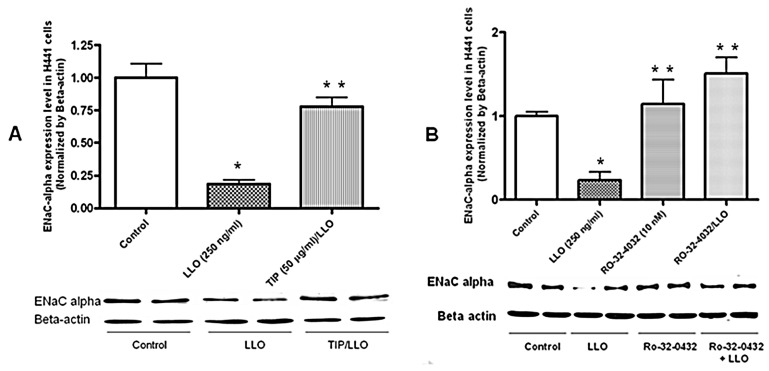
Pretreatment of H441 cells with the protein kinase C-α inhibitor Ro-32-0432 (10 nM) 30 min before LLO-treatment (4.3 nM) restores ENaC-α subunit expression at 2 h post LLO-treatment (4.3 nM). Mean ± SEM, *n* = 3, * *p* < 0.05 vs. ctrl; ** *p* < 0.05 vs. LLO.

Since LLO can activate PKC-α in H441 cells, an enzyme that can decrease ENaC expression in alveolar epithelial cells [33], we investigated the effect of the rather specific PKC-α/βII pharmacological inhibitor Ro-32-0432 on ENaC-α expression in LLO-treated H441 cells. As shown in Figure 3, a 30 min pretreatment of the cells with Ro-32-0432 (10 nM) significantly blunted the LLO-mediated reduction in ENaC-α protein expression in H441 cells at 2 h.

### 2.4. LLO Reduces Sgk-1 Phosphorylation at Residue T256 and Akt-1 Phosphorylation at Residue S473

**Figure 4:**
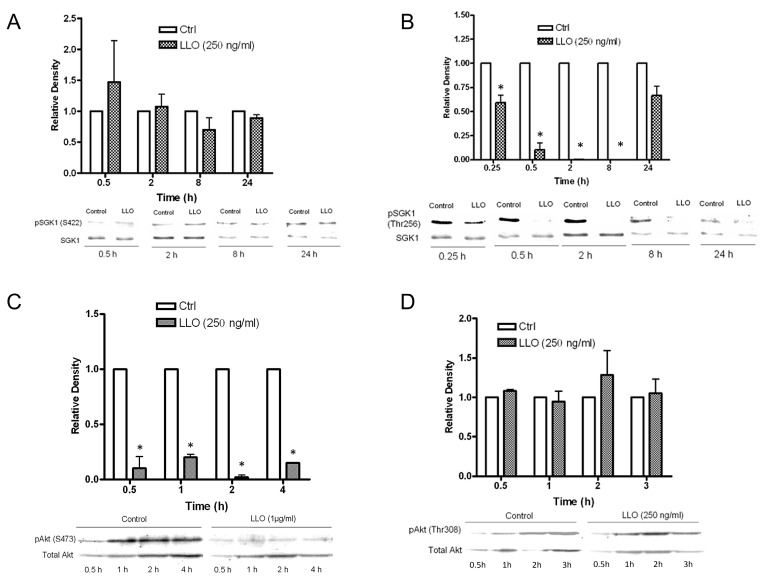
(**A**) Effect of LLO (4.3 nM) on p(S422)Sgk-1 expression (Western blotting) in H441 cells after 0.5, 2, 8, and 24 h of incubation. Mean ± SEM, *n* = 3, all groups: NS vs. control. (**B**) LLO (4.3 nM) blunts (T256)Sgk1 phosphorylation in H441 cells (Western blotting) after 0.25, 0.5, 2, and 8 h. This is partially restored at 24 h post-treatment. Mean ± SEM, *n* = 3, * *p* < 0.05 vs. ctrl. (**C**) LLO (250 ng/mL) blunts p(S473)Akt1 expression in H441 cells after 0.5, 1, 2, and 4 h of incubation. Mean ± SEM, *n* = 3, * *p* < 0.05 vs. ctrl. (**D**) Effect of LLO (250 ng/mL) on (T308)Akt1 phosphorylation in H441 cells after 0.5, 1, 2, and 3 h of incubation (Western blotting). Mean ± SEM, *n* = 3, all groups: NS vs. ctrl.

Since the previous experiments indicated that LLO activates a negative regulator of ENaC (i.e., PKC-α), here we assessed its effect on two positive regulators of the channel: Sgk-1 and Akt-1. Phosphorylation of Sgk-1 sequentially at residues S422 and T256 is required for full activation of the kinase [40,41]. Since Sgk-1 is a major positive regulator of ENaC expression, this phosphorylation pattern plays an important role in the ENaC-α subunit protein expression. We assessed phospho-Sgk-1-S422 and phospho-Sgk-1-T256 protein expression levels in H441 cells treated with or without 4.3 nM LLO at different time points, using immunoblot analysis. As demonstrated in Figure 4A, no significant differences could be observed between the control and LLO-treated groups regarding phospho-Sgk1-S422 expression from 0.5 h to 24 h. By contrast, as is shown in Figure 4B, LLO caused a significant reduction in phospho-Sgk-1-T256 expression, which could be observed from 0.25 h onward after LLO treatment and which continued for 24 h. The expression became essentially undetectable at the time points of 2 h and 8 h. After 24 h incubation, phospho-Sgk1-T256 expression was partially restored as compared to the control group. These data indicate that LLO significantly reduces the second phosphorylation level of Sgk-1 at residue T256.

Similar to Sgk-1, Akt-1 is also completely activated after phosphorylation at two key regulatory sites: T308 in the activation loop of the catalytic domain and S473 in the COOH-terminal domain. Since Akt-1 has also been implicated in the regulation of ENaC expression, we investigated the effect of LLO on total, phospho-T308, and phospho-S473 Akt-1 expression. As demonstrated in Figure 4C, LLO blunted the hydrophobic motif phosphorylation step at S473, required for the subsequent activation of Wnk1 (with no lysine kinase 1), which in turn promotes the phosphorylation of Sgk-1 at residue T256 [42]. By contrast, as shown in Figure 4D, LLO did not interfere with the T-loop phosphorylation step of Akt-1 at residue T308.

### 2.5. The TNF-Derived TIP Peptide Significantly Restores pT256Sgk-1 Expression Level at 24 h in LLO-Treated H441 Cells

**Figure 5:**
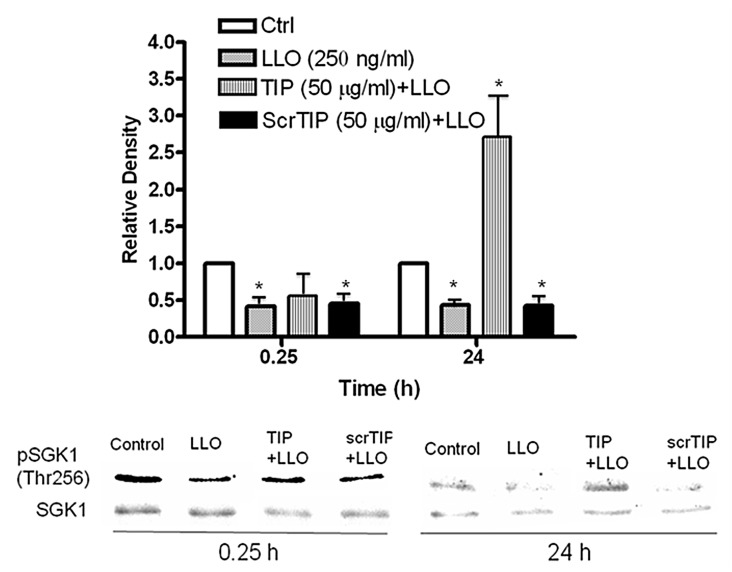
The TNF-derived TIP peptide, but not a scrambled TIP peptide (both at 50 μg/mL, 22 μM), restores LLO-blunted p(T256)Sgk-1 expression after 24 h of incubation when given 30 min prior to LLO (4.3 nM), and at that time point increases its expression significantly over the basal level. Mean ± SEM, *n* = 3, * *p* < 0.05 vs. ctrl.

Because the TNF-derived TIP peptide has been previously shown to activate alveolar liquid clearance in flooded mouse, rat, and rabbit lungs [28,29,30,31,32,33], we investigated whether it can also interfere with the LLO-induced downregulation of phospho-Sgk-1-T256 expression. Our results shown in Figure 5 indicate that the TIP peptide, but not a control scrambled TIP peptide (both at 22 μM), significantly restored phosphoSgk-1-T256 levels after 24 h of incubation, even above control levels, when given 30 min prior to LLO.

### 2.6. LLO Decreases Amiloride-Sensitive Sodium Current in H441 Cells

**Figure 6:**
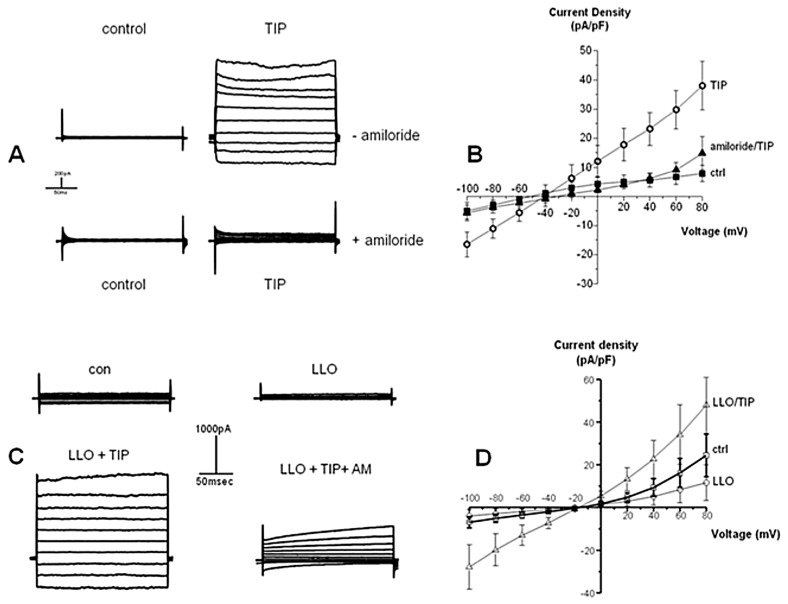
The TIP peptide increases amiloride-sensitive inward whole-cell current in H441 cells. Compared to the control group, the perforated-patch recordings show that the TIP peptide (50 µg/mL, 22 µM) significantly increases the inward whole-cell current in the absence of amiloride in H441 cells. The addition of amiloride (10 µM) into the recording solution significantly decreases the TIP peptide-induced inward whole-cell current. (**A**) Representative current trace; (**B**) Current-voltage plot (*n* = 7). Mean + SEM. * *p* < 0.05 vs. ctrl and vs. TIP + amiloride. The TIP peptide overrides LLO-induced inhibition of inward whole-cell current in an amiloride-sensitive manner in H441 cells. Compared to the control group, the perforated-patch recordings show that the treatment with LLO (4.3 nM) significantly decreases the inward whole-cell current, whereas the addition of the TIP peptide (22 µM) into the recording solution significantly recovers the inward whole-cell current in H441 cells, which is largely inhibited by 10 µM amiloride. (**C**) Representative current traces of cells treated with LLO, in the presence of TIP peptide or amiloride (AM); (**D**) Current-voltage plot of cells treated with LLO, in the presence or absence of TIP peptide. (*n* = 7). Mean + SEM of *n* = 6. * *p* < 0.05 vs. LLO.

Since our previous experiments had demonstrated that LLO activated PKC-α (a negative regulator of ENaC) and blunted the activity of Sgk-1 and Akt-1 (two positive regulators of ENaC activity), we investigated the effect of the exotoxin on the amiloride-sensitive sodium currents in H441 cells, which is a good indicator of ENaC function. To that purpose, we performed whole-cell perforated-patch clamp recordings, which provide a more accurate means of measuring whole-cell currents than standard whole-cell techniques. Cells were pretreated or not for 30 min with 22 μM TIP peptide with/without the presence of 10 µM amiloride in the recording solution, prior to incubation with LLO. As shown in Figure 6A,B, the TIP peptide increased inward whole-cell current in an amiloride-sensitive manner in H441 cells. As shown in Figure 6C,D, perforated-patch recordings show that LLO (4.3 nM) significantly decreased the inward whole-cell current in H441 cells, which is significantly prevented upon the administration of the TIP peptide, which in turn upregulates sodium uptake above basal levels to attain the same level as in the absence of LLO. Again, subsequent amiloride treatment can inhibit the TIP peptide-induced recovery in inward whole-cell current in H441 cells. These results demonstrate that the TIP peptide can prevent the LLO-induced decrease in amiloride-sensitive inward current in H441 cells and indicate that the peptide blunts LLO-mediated ENaC dysfunction.

### 2.7. The TNF-Derived TIP Peptide Reduces Edema Formation in LLO-Treated C57BL/6 Mice

**Figure 7:**
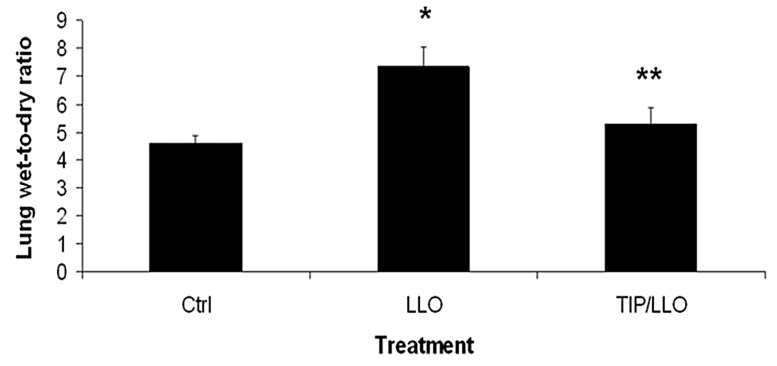
The TIP peptide (2.5 mg/kg) inhibits the LLO-induced increase in lung wet-to-dry ratio in male C57BL/6 mice, 6 h after intratracheal LLO instillation (12.5 μg/kg). Mean ± SEM, *n* = 7, * *p* < 0.05 vs. ctrl; ** *p* < 0.05 vs. +TIP.

In order to investigate whether LLO also dysregulates ENaC function and thus edema reabsorption in mice in vivo, we have treated male C57BL/6 mice with 12.5 μg/kg LLO in the presence or absence of 2.5 mg/kg of the TIP peptide (*n* = 7 per group). As shown in Figure 7, intratracheal instillation of LLO provoked significant edema formation, as assessed by the significantly increased wet-to-dry ratio of the isolated lungs after 6 h. Co-treatment of the mice with the TIP peptide significantly blunted the edema-promoting activity of LLO.

## 3. Discussion

Pulmonary edema is a pathological dysbalance between fluid extravasation and fluid reabsorption in the alveoli and capillaries. Apically expressed ENaC in type I and II alveolar and small airway epithelial cells promotes fluid absorption in the alveoli and small airways. Therefore, impaired ENaC function can cause lung fluid accumulation, contributing to the formation of pulmonary edema [6]. Listeriolysin is a prototypical member of the cholesterol-dependent cytolysin family of pore-forming proteins produced by many Gram-positive organisms [4,5]. Especially in neonates, immune-suppressed individuals, and pregnant women, *Listeria* can invade the lungs where it can lead to potentially lethal pulmonary edema formation. In our study, LLO induced pulmonary edema within 6 h upon intra-tracheal instillation of as little as 12.5 μg/kg in mice. As LLO is known to disrupt barrier function, an understanding of its effects on ion transporters involved in alveolar sodium uptake and edema reabsorption is clinically highly relevant.

We investigated whether LLO can affect the expression and function of ENaC in the well-characterized human airway epithelial cell line H441. We should note that our experimental approach has limitations since (a) H441 monolayers at an air-liquid interface represent a more physiological condition than studying these cells in culture and (b) culture conditions of H441 cells may influence whether they express ENaC [43,44]. We have, however, demonstrated in this and a recently published study [34] that the H441 cells we have used express all three ENaC subunits and, moreover, display amiloride-sensitive Na^+^ currents in patch-clamp measurements.

Our results indicate that LLO impairs the expression of both the uncleaved and cleaved mature α subunit of ENaC and of ENaC-β in H441 cells, especially the former of which is crucial for the channel’s activity [8]. This LLO effect involves Ca^2+^-mediated activation of PKC-α, characterized by the increased phosphorylation and translocation of the enzyme to the plasma surface membrane. The specific PKC-α inhibitor Ro-32-0432 and the TNF-derived TIP peptide both blunt LLO-mediated PKC-α activation and were able to completely prevent the deleterious effect of LLO on ENaC expression. It is interesting to note that although they induce completely different signaling pathways in alveolar epithelial cells, both LPS and LLO seem to depend on PKC-α activation for their effects on ENaC expression [22], making this enzyme an attractive target for novel therapies addressing pulmonary edema formation during both Gram-negative and Gram-positive bacterial infections. It should be noted, however, that the effect of LLO on ENaC expression, although indirect, is not necessarily only an epiphenomenon of the toxin’s capacity to induce Ca^2+^ influx. Indeed, the mere increase in intracellular Ca^2+^ concentrations does not always lead to ENaC inhibition. As a matter of fact, Ca^2+^ ionophore-induced increases in intracellular Ca^2+^ were shown to activate rather than inhibit the expression of all three subunits and, moreover, activated the positive ENaC regulator Sgk-1 under hypotonic conditions in A6 cells [45]. Moreover, our observation that LLO decreased the expression of prostasin, an enzyme crucial in the activation and maturation of ENaC subunits [35], indicates that the toxin can specifically interact with regulators of the channel.

Apart from PKC-α, the expression of ENaC at the cell membrane surface is also negatively regulated by the E3 ubiquitin protein ligase, Nedd4-2, which causes ubiquitination-dependent internalization of ENaC [11]. Positive regulators of ENaC such as Sgk-1 and Akt-1 inhibit the action of Nedd4-2, thereby upregulating ENaC expression and activity. Maximal stimulation of Sgk-1 requires the mTORC2-mediated phosphorylation at S422 in the hydrophobic motif at its COOH terminus and the phosphoinositide-dependent kinase 1 (PDK1)-mediated phosphorylation at T256 within the activation loop (T-loop) [41]. Prior phosphorylation of Sgk1 at the hydrophobic motif (S422) promotes its subsequent interaction with the PDK1 interacting fragment (PIF)-binding pocket of PDK1 and its T-loop phosphorylation (at T256) [40]. Recent studies suggest that WNK1 (with no lysine kinase 1), which is a substrate of Akt-1, cooperates with PDK1 to activate Sgk-1, leading to activation of ENaC [42].

Interestingly, LLO significantly reduced the phosphorylation level of Sgk-1 at the T256 but not at the S422 residue. Since WNK1, which mediates the T-loop phosphorylation step of Sgk-1, is activated by Akt-1, we have also investigated the effect of LLO on the activation of this kinase. Our data demonstrate that LLO significantly inhibits Akt-1 activation, since the phosphorylation at residue S473, but not at residue T308, is blunted. Taken together, our findings indicate that LLO inhibits the S473 phosphorylation step in Akt1 and the T256 phosphorylation step in Sgk1. Since PDK1 was reported to mediate both the phosphorylation of T308 in Akt-1, which is not inhibited by LLO, as well as of T256 in Sgk1, which is blunted by the toxin, these data preclude PDK1 as a target for LLO. Since both Akt1 and Sgk1 are positive regulators of ENaC expression, the consequence of these events will be a reduced expression of this channel, as we could demonstrate here. We should, however, note here that the effects of LLO on Sgk-1 and Akt-1 activation occur at later time points than that observed on PKC-α activation. Moreover, the TNF-derived TIP peptide, which upregulates sodium uptake in H441 cells both in the presence and in the absence of LLO, is able to restore the activation status of Sgk-1, but not Akt-1, and then only at a much later time point than its effects on PKC-α activation and sodium uptake. This points toward a more important role for the activation of the negative regulator of ENaC (i.e., PKC-α) than for the reduced activity of its positive regulators Sgk-1 and Akt-1 by LLO in these cells.

Although TNF decreases amiloride-sensitive current as well as the steady-state mRNA and protein levels of ENaC in adult alveolar epithelial cells [18], the cytokine has also been shown to increase alveolar fluid clearance in a rat pneumonia model [46]. Spatially distinct from its receptor binding sites, TNF also harbors a lectin-like domain [23,24], which can be mimicked by the 17 AA TIP peptide [24]. The TIP peptide increases Na^+^ uptake in alveolar epithelial cells upon binding to the α subunit [31,33,34]. In order to investigate whether the TIP peptide can also promote sodium uptake in alveolar epithelial cells under conditions of bacterial infection, we have tested here its capacity to interfere with LLO-mediated suppression of ENaC expression and function. Our results demonstrate that the TIP peptide blunts PKC-α activation, restores pT256Sgk1-expression, increases ENaC-α protein expression, and restores ENaC function in the presence of LLO in H441 cells. Moreover, the peptide prevents edema formation upon intratracheal instillation of LLO in mice. An interesting observation is that the TIP peptide does not significantly increase ENaC expression in H441 cells, but it does significantly increase Na^+^ uptake. Since ENaC activity is the product of its expression level *N* at the cell surface and its open probability time *Po*, this indicates that the peptide increases ENaC open probability, as we could demonstrate in recent studies [33,34].

Another observation is that the TIP peptide apart from increasing inward Na^+^ currents, also activated outward currents. This might be the consequence of activating not just classical ENaC but also the recently discovered hybrid ENaC-α/acid-sensing ion channel 1a (ASIC1a) non-selective cation channels [47]. These NSC channels have a higher conductance than ENaC and display a more linear current pattern [48]. NSC channels consisting of ENaC-α and ASIC1a were recently shown to at least partially mediate the barrier protective activities of the TIP peptide in capillary lung endothelial cells [49].

The observed protective effect of the TIP peptide in LLO-induced permeability edema in mice is likely a combination of the previously reported capillary endothelial barrier protection [50] and a stimulation of alveolar liquid clearance [33]. As such, it is unclear at this point what the contribution is of improved alveolar fluid clearance versus improved barrier function in the in vivo effect of the peptide. Since both NSC and ENaC are involved in both of these activities, the role of ENaC, which is more sensitive to amiloride, could be possibly assessed using a low dose treatment (1 μM). These data indicate that the peptide might represent an attractive therapeutic agent to treat *Listeria monocytogenes* infection-associated lung injury. In a recent clinical trial, the TIP peptide was shown to significantly increase lung liquid clearance in patients with acute lung injury having a sequential organ failure assessment (SOFA) score of 11 or higher [51]. The SOFA score is a scoring system that determines the extent of organ function or its rate of failure and is based on six different scores, one each for the cardiovascular, respiratory, hepatic, coagulation, renal, and neurological systems. These results indicate that the TIP peptide has protective effects on ENaC expression and the restored Sgk-1 phosphorylation at T256 residue might be involved in this effect. In view of the important physiological activating role of aldosterone in ENaC regulation [52], future studies assessing the effect of LLO and of other pore-forming toxins on its expression will be interesting. Moreover, effects of LLO on internalization, recycling, and ubiquitination pathways seem warranted [53,54]. 

The complex physiology of eukaryotic cells is regulated by a number of multilayered and interconnected mechanisms. Transcription of new mRNA, alternative RNA splicing, and translation into a protein that can be further modified by select post-translational alterations create a continuously fine-tuned regulatory network [55]. Some studies have shown that *Listeria* can manipulate the host cell through its important effects on cellular post-translational modification. Through its major exotoxin LLO, *Listeria monocytogenes* induces a dramatic dephosphorylation of histone H3 as well as a deacetylation of histone H4 during early phases of infection [56]. LLO was shown to affect SUMOylation, an essential post-translational modification in eukaryotic cells [57].

In this study, we have demonstrated that LLO can modify the phosphorylation of Sgk-1, Akt-1, and PKC-α at specific residues with major consequences for the function of ENaC and thus for the alveolar liquid clearance capacity, which is crucially dependent on vectorial Na^+^ transport. These data suggest the importance of post-translational regulations in the complex mechanisms underlying the pathogenesis of *Listeria monocytogenes* in the lungs.

## 4. Conclusions

LLO significantly impairs ENaC activity, at least partially, by activating PKC-α (a negative regulator of ENaC expression and open probability) and by blunting the activation of two positive regulators of ENaC activity (Sgk-1 and Akt-1). These activities of the toxin contribute to pulmonary edema formation upon intratracheal instillation into mice. The TNF-derived TIP peptide, which was previously shown to protect from LLO-induced endothelial barrier dysfunction [50], preserves mature ENaC-α expression and ENaC activity in the presence of LLO, thus favoring its further therapeutic evaluation in patients with G^+^ pneumonia.

## 5. Materials and Methods

### 5.1. Cells

The human bronchiolar epithelial cell line H441, purchased from the American Type Culture Collection (Manassas, VA, USA), was derived from the pericardial fluid of a patient with papillary adenocarcinoma of the lung. Cells were cultured in RPMI-1640 medium (Mediatech Inc., Manassas, VA, USA) with 10% fetal bovine serum (HyClone, Logan, UT, USA) and incubated at 37 °C in an atmosphere of 95% O_2_–5% CO_2_. The medium was replaced every other day and cultures were split three times a week in a sub-cultivation ratio of 1:10 using Trypsin/EDTA (Sigma-Aldrich, St. Louis, MO, USA). For immunoblotting experiments, H441 cells were cultured in flasks and then split into 6-well cell culture plates (Fisher, Suwanee, GA, USA).

### 5.2. Animals

Eight-week-old male C57BL/6 wild-type mice (*n* = 21, 20–22 g) were purchased from Charles River Laboratories (Raleigh, NC, USA). These animals were housed in air-filtered, temperature-controlled units with food and water. All procedures were approved by the Institutional Animal Research Committee of the Medical College of Georgia at Augusta University. (BSP#0755, 25 October 2017).

### 5.3. Peptides

Human TNF TIP peptide and scrambled TIP peptide, as acetate salt, were purchased from Ambiopharm, North Augusta, SC, USA). The amino acid sequences were:

- human TIP (hTIP) peptide: CGQRETPEGAEAKPWYC
- scrambled TIP peptide: CGTKPWEAGEQERPAYC

Peptides were made cyclic through CC oxidation.

### 5.4. Chemicals and Antibodies

Molecular weight standards, acrylamide, SDS, *N*,*N*′-methylene-bisacrylamide, *N*,*N*,*N*′,*N*′-tetramethylenediamine, protein assay reagents, and nitrocellulose membranes were purchased from Bio-Rad Laboratories (Hercules, CA, USA). Protein kinase C inhibitor Ro-32-0432 was from Calbiochem (La Jolla, CA, USA). Rabbit polyclonal anti-prostasin antibodies, anti-phospho-Sgk-1 (S422) and anti-total-Sgk-1 antibodies were obtained from Abcam Inc. (Cambridge, MA, USA). The anti-Sgk-1 (T256) antibody, the rabbit anti-total human Akt polyclonal antibody, as well as mouse anti-human phospho-Akt (S473) and anti-human phospho-Akt (T308) mAb were purchased from Cell Signaling (Danvers, MA, USA). Anti-beta-ENaC antibodies were purchased from ProteinTech (Rosemont, IL, USA) and anti-gamma-ENaC antibodies were from Abcam (Cambridge, MA, USA). Rabbit polyclonal anti-human ENaC-alpha antibodies were either made in-house (ENaC-alpha 60) [35] or were purchased from Novus Biologicals (Littleton, CO, USA). The Pierce supersignal substrate chemiluminescence detection kit was obtained from Pierce (Rockford, IL, USA). Monoclonal anti-β-actin antibody, fibronectin, Tween-20, and all other chemicals were purchased from Sigma-Aldrich (St. Louis, MO, USA).

### 5.5. Purification of LLO

LLO was expressed and purified from the wild-type *L. innocua* 6a strain. A 10 mL volume of an overnight bacterial culture grown at 37 °C in BHI broth was used to inoculate one liter of the chemically defined minimal medium. Following 48 h incubation at 30 °C, bacteria were removed by centrifugation and the supernatant fluid was concentrated to 50 mL using a Millipore filtration apparatus with a cut-off point of 10 kD. The crude supernatant of LLO was then batch absorbed for 60 min with Q-Sepharose or SP-Sepharose (Pharmacia, Freiburg, Germany) and pre-equilibrated with loading buffer (50 mM NaH_2_PO_4_, pH 6.2). The non-absorbed fraction was centrifuged and desalted by transferring through a super loop to a HiPrep 26/10 desalting column (Pharmacia, Freiburg, Germany) where loading buffer (50 mM NaH_2_PO_4_, pH 6.2) was used to elute the desalted fraction. This fraction was subsequently filtered through a Millipore filter (0.22 µM) and loaded onto a Resource-S column previously equilibrated with 50 mM NaH_2_PO_4_, pH 6.2. The pure toxin eluted reproducibly from the column at 0.21 to 0.28 M NaCl using an elution buffer (50 mM NaH_2_PO_4_, 1 M NaCl, pH 5.6). Fractions were collected and individually tested for hemolytic activity. Yields of the toxins ranged from 1 to 5 mg/L supernatant with a hemolytic activity (HU) of 20,000 HU/mg purified protein. One hemolytic unit (HU) is expressed as the amount of toxin required to lyse 50% of a 1% suspension of sheep erythrocytes. It should be noted that storage of the toxin, even at −80 °C, can result in a partial loss of hemolytic activity. As such, we have used varying concentrations of the toxin in the different experiments. Protein desalting and purification processes were carried out using the high performance chromatography system ÄKTA explorer and UNICORNTM control system (Pharmacia, Freiburg, Germany). The toxin showed a high purity in SDS-PAGE, was efficiently recognized with LLO-specific antibodies, and exhibited hemolytic activity on sheep erythrocytes at both pH 6.0 and pH 7.4. Protein concentrations were determined using a standard assay (Bio-Rad Protein Assay; Bio-Rad, Munich, Germany).

### 5.6. Immunoblotting

After the different treatments, cells were first washed twice with ice-cold phosphate buffered saline (PBS) containing sodium orthovanadate (1 mM); then cells were incubated with 400 μL of ice-cold modified RIPA lysis buffer (20 mmol/L Tris pH 7.4, 150 mmol/L NaCl, 1 mmol/L EDTA, 1 mmol/L EGTA, 1% Triton X-100, 2.5 mmol/L sodium pyrophosphate, 1 mmol/L β-glycerophosphate, 1 mmol/L Na_3_VO_4_, 1 μg/mL leupeptin, and 1 mmol/L phenylmethylsulfonyl fluoride) for 5 min on ice. The lysed cells were then scraped off and sonicated (3 times for 5 s each) on ice. After centrifugation for 10 min at 20,000× *g* at 4 °C, the protein concentration in the supernatant was determined using BCA™ Protein Assay Kit (Pierce, Rockford, IL, USA). Bovine serum albumin (Pierce, Rockford, IL, USA) was used as a standard. Each measurement of protein concentration was performed in duplicate. Cell lysates were placed in denatured SDS sample buffer and boiled for 5 min and loaded onto either 5%, 7.5%, or 10% polyacrylamide minigel (Bio Rad Laboratories, Hercules, CA, USA) depending on the different molecular weight. Proteins were separated by electrophoresis at 75 V in stacking gel and 150 V in resolving gel. Molecular weight markers were electrophoresed simultaneously with samples. The gels were then transferred to nitrocellulose membranes in a transfer buffer (50 mmol/L Tris-HCl pH 7.0, 380 mmol/L glycine, and 20% methanol). After blocking with 5% non-fat dry milk dissolved in TTBS (TBS with 0.1% Tween 20) at room temperature for 1 h, the membranes were incubated with different antibodies overnight at 4 °C. Subsequently, nitrocellulose membranes were washed twice for 10 min each with TTBS and incubated with horseradish peroxidase-conjugated IgG antibody (Cell Signaling, Danvers, MA, USA) for 1 h at 37 °C. The blots were developed with an enhanced chemiluminescence detection kit (Pierce Biotechnology, Rockford, IL, USA) according to the manufacturer’s instructions. Then a diagnostic film (Kodak, Rochester, NY, USA) was exposed to the membrane. The blot densities were analyzed with an NIH Imager (Scion, Frederick, MD, USA). Bound antibodies were removed from Western blot membranes by incubation for 30 min at room temperature in Restore Western Blot Stripping Buffer (Pierce, Rockford, IL, USA). After washing the membranes 3 times for 10 min with TBS-T, they were ready for another immunodetection of proteins.

### 5.7. Surface Biotinylation

H441 cells were washed twice with ice-cold PBS and biotinylated using the Pierce cell surface protein isolation kit (Thermo Scientific, Grand Island, NY, USA) according to the manufacturer’s instructions. Cells were then trypsinized and collected by centrifugation (500 g for 3 min). After lysing the cells with the provided lysis buffer, the samples were subjected to immunoprecipitation. The clarified supernatants were incubated with 2 µg of ENaC-α, ENaC-β, or ENaC-γ antibody for 2 h at 4 °C on a head-overhead shaker and then added to the equilibrated protein G magnetic beads (Invitrogen, Carlsbad, CA, USA) to further incubate overnight at 4 °C on a head-overhead shaker. The protein G complexes were washed and disrupted with 1% SDS in lysis buffer for 80 min at 37 °C. The beads were then separated on a magnet, and the supernatant was used for the isolation of biotin-labeled proteins, according to the manufacturer’s protocol. Briefly, the NeutrAvidin-agarose was transferred to the provided columns and equilibrated, and the samples were incubated with this slurry for 3 h at 4 °C on a head-overhead shaker. After washing the columns three times with wash buffer, biotinylated proteins were eluted with hot Laemmli buffer containing 50 mM DTT. Eluted proteins were subjected to SDS-PAGE and Western blotting with anti-ENaC-α 60 polyclonal antibodies, as described previously [35] or with commercially available anti-human ENaC-α, β, or γ polyclonal Ab (Novus Biologicals, Littleton, CO, USA).

### 5.8. Measurement of PKC-α Activation in H441 Cells

Unless otherwise indicated, all steps were conducted at 4 °C or on ice using chilled buffers. After incubation with LLO (1 µg/mL, 17.2 nM) in the presence or absence of TIP peptide (50 µg/mL, 22 µM), H441 cells were first washed twice with ice-cold phosphate buffered saline (PBS) and then incubated for 10 min with 1–2 mL isotonic MSE buffer consisting of 10 mM Tris-HCl, pH 7.5, 220 mM mannitol, 70 mM sucrose, 1 mM EGTA, 0.025% fatty acid-free bovine serum albumin (BSA), 1.6 mM carnitine, 2 mM taurine, and 10 µg/mL each of aprotinin, leupeptin, and phenylmethylsulfonyl fluoride. Subsequently, the cells were collected using cell scrapers, transferred to glass tubes and subjected to homogenization using a motorized homogenizer. The homogenate was transferred to Eppendorf tubes and centrifuged at 11,000× *g* for 10′ at 4 °C and the supernatant from this spin was then subjected to a 100,000× *g* centrifugation for 20′ at 4 °C in order to obtain the membrane fraction pellet. This pellet was then resuspended in 100 µL of MSE buffer. The protein concentrations of the resuspended and supernatant samples were determined using BCA™ Protein Assay Kit (Pierce, Rockford, IL, USA). Same amounts of samples were placed in denaturing SDS sample buffer, boiled for 5 min, and loaded onto a 7.5% polyacrylamide minigel (Bio Rad Laboratories, Hercules, CA, USA). Proteins were separated by electrophoresis at 75 V in stacking gel and 100 V in resolving gel. The gels were then transferred to nitrocellulose membranes in a transfer buffer (50 mmol/L Tris-HCl pH 7.0, 380 mmol/L glycine, and 20% methanol). After being blocked for 1 h at room temperature in 1% BSA buffer dissolved in TTBS (TBS with 0.1% Tween 20), the membranes were incubated overnight with purified mouse anti-human PKC-α antibodies (all from BD Transduction, Lexington, KY, USA). Subsequently, nitrocellulose membranes were washed three times for 10 min each with TTBS and incubated with the appropriate secondary antibody (Amersham, Arlington Heights, IL, USA). The luminescence detection of peroxidase was performed with the enhanced chemiluminescence system (ECL; Amersham, Arlington Heights, IL, USA) according to the manufacturer’s instructions and diagnostic films (Kodak, Rochester, NY, USA) were exposed to the membranes. The blot densities were analyzed with an NIH Imager (Scion, Frederick, MD, USA).

### 5.9. Whole Cell Patch Clamp Recording

Whole-cell currents were measured from metabolically intact H441 cells by using the perforated-patch technique. In contrast to standard whole-cell techniques, perforated-patch recordings provide accurate current measurement with only minimal current decay or loss of soluble cytoplasmic components due to cellular dialysis. Furthermore, endogenous calcium buffering is not inactivated by dialyzing cells with calcium chelators, as are required during whole-cell recordings. Thus, the perforated-patch technique provides a more accurate means of measuring whole-cell currents. H441 cells were grown on 12 mm cover slips and placed in a recording solution of the following composition: 140 mM Na acetate, 5 mM KOH, 2 mM MgSO_4_, 10 mM HEPES, 10 mM glucose (pH 7.2). Both calcium and chloride were reduced in this solution to attenuate currents resulting from the flux of these ions. In most experiments 2 mM CdCl_2_ was also included in the extracellular solution to limit the potential influence of currents carried via non-selective cation channels. Amiloride (10 μM) was used to inhibit ENaC currents. Patch pipettes with a resistance of 3 MΏ or less were fabricated from capillary tubes using a P-2000 laser pipette puller (Sutter Instrument Co., Novato, CA, USA). To help isolate Na^+^ currents, the tip of the patch pipette was filled with a solution containing 130 mM Cs_2_SO_4_, 5 mM CsOH, 2 mM CaCl_2_, 2 mM MgCl_2_, and 10 mM HEPES (pH 7.2). Cesium diffuses through the membrane perforations and blocks K channels from the cytoplasmic surface of the membrane. The remainder of the pipette was back-filled with a similar solution to which 200 µg/mL amphotericin B (diluted by sonication from a 50 mg/mL stock in dimethyl sulfoxide) was added. Cells were studied only if the voltage drop across the series resistance was reduced to <5 mV within 10–20 min after forming a giga-Ώ seal. Voltage clamp and voltage pulse generation were controlled with an Axopatch 200B patch-clamp amplifier (Axon Instruments, Inc., Sunnyvale, CA, USA) and data were analyzed with pCLAMP 10.0 (Molecular Devices Axon Instruments, Inc., San Jose, CA, USA). Currents were filtered at 2 kHz and digitized at 10 kHz. In order to control for variation in cell size, normalized plots of current density (cellular current/cellular capacitance) versus voltage were constructed for analysis. All substances were diluted into fresh bath solution and perfused into a 1.0 mL recording chamber (Warner Instruments Corp., Holliston, MA, USA).

### 5.10. Determination of the Lung Wet-to-Dry Weight Ratio

Male C57BL/6 mice of 20 g were anesthetized with intraperitoneal ketamine (150 mg/kg) and acetylpromasine (15 mg/kg). Then the trachea and the right internal jugular vein was exposed via neck and chest incisions, respectively. LLO in the presence or absence of TIP peptide or sterile saline was instilled intratracheally via a 20-gauge catheter. Simultaneously, mice received TIP peptide (2.5 mg/kg) or saline (in the control group). The animals were allowed to recover for 6 h and were then sacrificed. The lungs are collected and stored at −70 °C for evaluation of lung injury. Three groups of C57BL/6 mice were given the following treatments: Group 1: seven male C57BL/6 mice were injected intratracheally with 25 μL saline/mouse; Group 2: seven male C57BL/6 mice were injected intratracheally with 250 ng LLO/mouse (dissolved in 25 μL saline); Group 3: seven male C57BL/6 mice were co-treated with 50 µg human TIP peptide and 12.5 μg/kg LLO (dissolved in 25 μL saline). After 6 h, animals were terminally narcotized with 100 μL ketamin/xylazin (1.3:1) i.p. and the heart–lung block was excised. Adjacent blood or tissue was removed carefully. Lung lobes were removed and the wet weight was determined. Lungs were subsequently dried in an oven at 60 °C for 48 h and the wet-to-dry weight ratio was calculated.

### 5.11. Data Analysis

Statistical analysis was performed using GraphPad Prism 4 (GraphPad Software, La Jolla, CA, USA). All data were expressed as mean ± SE. Statistical significance between two groups was evaluated by the Student’s t-test for paired data. Comparison among multiple groups was made by using one-way ANOVA for multiple comparisons. A *p* value < 0.05 was considered significant.
